# Single‐ Versus 2‐Stent Strategies for Coronary Bifurcation Lesions: A Systematic Review and Meta‐Analysis of Randomized Trials With Long‐Term Follow‐up

**DOI:** 10.1161/JAHA.118.008730

**Published:** 2018-05-25

**Authors:** Thomas J. Ford, Peter McCartney, David Corcoran, Damien Collison, Barry Hennigan, Margaret McEntegart, David Hildick‐Smith, Keith G. Oldroyd, Colin Berry

**Affiliations:** ^1^ West of Scotland Heart and Lung Centre Golden Jubilee National Hospital Glasgow United Kingdom; ^2^ British Heart Foundation Glasgow Cardiovascular Research Centre Institute of Cardiovascular and Medical Sciences University of Glasgow United Kingdom; ^3^ University of New South Wales Sydney Australia; ^4^ Division of Cardiology Royal Sussex County Hospital Brighton United Kingdom

**Keywords:** bifurcation, bifurcation intervention, bifurcation lesion, provisional stenting, Percutaneous Coronary Intervention, Revascularization, Stent, Mortality/Survival, Meta Analysis

## Abstract

**Background:**

The majority of coronary bifurcation lesions are treated with a provisional single‐stent strategy rather than an up‐front 2‐stent strategy. This approach is supported by multiple randomized controlled clinical trials with short‐ to medium‐term follow‐up; however, long‐term follow‐up data is evolving from many data sets.

**Methods and Results:**

Meta‐analysis of randomized controlled trials evaluating long‐term outcomes (≥1 year) according to treatment strategy for coronary bifurcation lesions. Nine randomized controlled trials with 3265 patients reported long‐term clinical outcomes at mean weighted follow‐up of 3.1±1.8 years. Provisional single stenting was associated with lower all‐cause mortality (2.94% versus 4.23%; risk ratio: 0.69; 95% confidence interval, 0.48–1.00; *P*=0.049; I^2^=0). There was no difference in major adverse cardiac events (15.8% versus 15.4%; *P*=0.79), myocardial infarction (4.8% versus 5.5%; *P*=0.51), target lesion revascularization (9.3% versus 7.6%; *P*=0.19), or stent thrombosis (1.8% versus 1.6%; *P*=0.28) between the groups. Prespecified sensitivity analysis of long‐term mortality at a mean of 4.7 years of follow‐up showed that the provisional single‐stent strategy was associated with reduced all‐cause mortality (3.9% versus 6.2%; risk ratio: 0.63; 95% confidence interval, 0.42–0.97; *P*=0.036; I^2^=0).

**Conclusions:**

Coronary bifurcation percutaneous coronary intervention using a provisional single‐stent strategy is associated with a reduction in all‐cause mortality at long‐term follow‐up.

**Clinical Perspective**

**What Is New?**

- This large meta‐analysis of randomized controlled trials of bifurcation coronary stent strategies supports a provisional single‐stent strategy over upfront 2‐stent strategy.

**What Are the Clinical Implications?**

- Coronary bifurcation angioplasty may be performed safely using a provisional single‐stent approach.
- This simpler option, as appropriate, may reduce fatal long‐term sequelae associated with complex 2‐stent bifurcation strategies.

Bifurcation lesions are common and account for up to one fifth of percutaneous coronary interventions. Typically, they are defined as a lesion at or near a significant division of a major epicardial artery into 2 branches (main vessel [MV] and side branch [SB]).^1^ This patient group merits special attention because of the high burden of adverse events following treatment.^2^ Many clinical trials have compared a single‐stent strategy (MV only with a provisional approach to SB stenting) with an up‐front 2‐stent strategy. At short‐term follow‐up, randomized controlled trials (RCTs) show overall similar efficacy between the 2 approaches; however, a provisional single‐stent strategy (with bailout use of a second stent) demonstrates improved safety and lower costs.^3^ Recent data emerging from Asia support the double‐kissing (DK) crush 2‐stent technique over provisional stenting, refueling the debate about the optimal treatment of these lesions.^4^, ^5^

A short‐term focus on these patients will miss detection of important late complications (>1 year) after bifurcation percutaneous coronary intervention including death, myocardial infarction (MI), stent thrombosis (ST), and target lesion revascularization (TLR). These important events may accrue particularly as the protective effect of dual antiplatelet therapy (DAPT) is withdrawn, unmasking sequelae of underexpanded stents and malapposed struts. The frequency of these outcomes may vary in the longer term, according to treatment strategy. Consequently, we performed a meta‐analysis of RCTs comparing the long‐term clinical outcomes of 1‐ and 2‐stent strategies for treating coronary bifurcation lesions.

## Method

The data, analytic methods, and study materials have been made available to other researchers for the purposes of reproducing the results or replicating the procedure. The full study protocol was registered with the PROSPERO international database of prospectively registered systematic reviews in health and social care (CRD42017081091). This study was performed following the PRISMA (Preferred Reporting Items for Systematic Reviews and Meta‐Analyses) guidelines.^6^

### Eligibility Criteria

The inclusion criteria for studies were as follows: clinical RCTs comparing provisional versus up‐front 2‐stent strategies for bifurcation lesions regardless of the specific stenting technique and SB size and lesion complexity. Publications were excluded if they compared various 2‐stent techniques without a provisional stenting arm, utilized a dedicated bifurcation stent or included non–coronary bifurcation lesions. Nonrandomized trials, publications not in English, and those without clinical outcomes of interest during at least 1 year of follow‐up were all excluded.

### Quality Assessment

**Figure 1:**
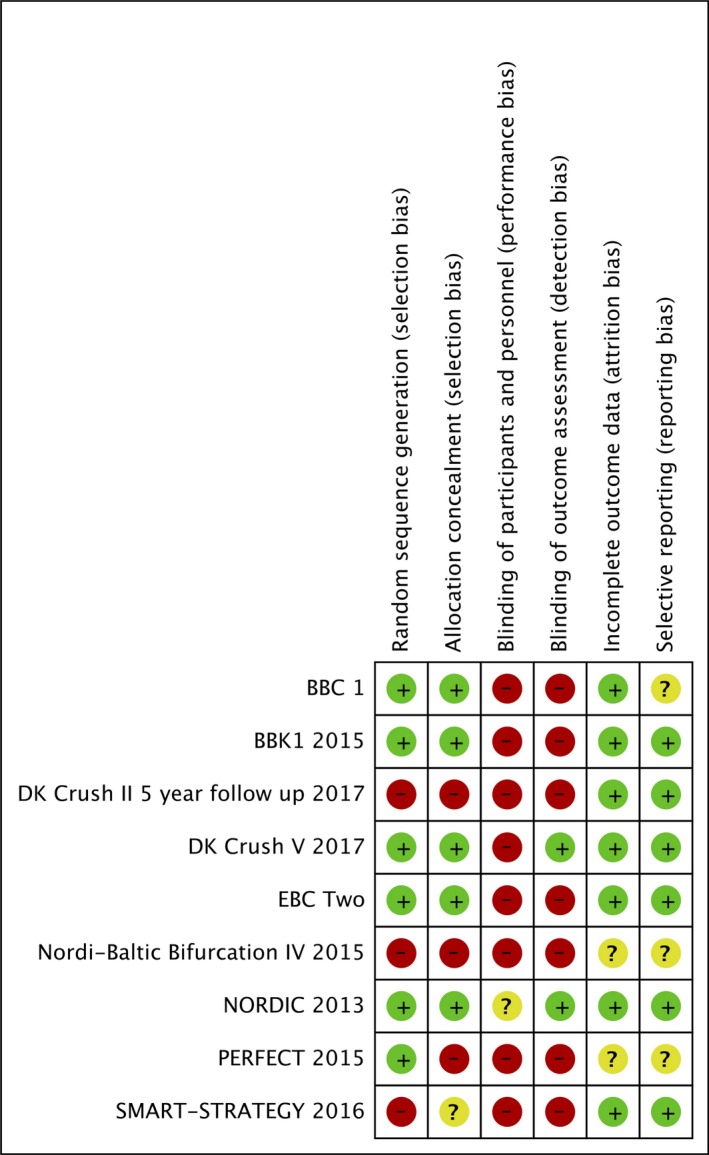
Risk of bias summary—Cochrane risk tool.

Risk of bias and quality assessment of the included trials was independently performed by 2 reviewers (T.F. and P.M.) Discrepancies were resolved by consensus with a third independent reviewer (D.C.). Quality assessment according to the Cochrane Collaboration framework is provided (Figure 1).^7^ Publication bias was assessed according to funnel plot asymmetry using standard error as the measure of study size and risk ratio of treatment effect.^8^

### Search Strategy

A systematic search of the online databases PubMed, Cochrane Central Register of Controlled Trials (CENTRAL), EBSCO (Medline), and Web of Science was performed through November 11, 2017. Peer‐reviewed RCTs were selected using combinations of the keywords *bifurcation, coronary, outcomes* and *provisional*. Two reviewers (T.F. and P.M.) independently screened abstracts against inclusion and exclusion criteria, and reference lists of relevant articles and meta‐analyses were reviewed for other relevant studies. Conference abstracts from recent major cardiology meetings were searched for completeness, including EuroPCR, the European Society of Cardiology, the American College of Cardiology, the American Heart Association, and Transcatheter Cardiovascular Therapeutics.

### Outcomes and Definitions

The primary outcome was all‐cause mortality. Secondary outcomes included MI, definite or probable ST, TLR, and major adverse cardiac events (MACE). Outcome data were extracted by 2 independent authors (T.F and D.C.). Differences in opinion were resolved by consensus involving a third reviewer (P.M.). Probable or definite ST was determined according to the Academic Research Consortium definition.^9^ MACE was defined as the composite of death, MI, and target vessel revascularization (TVR). Minor variations in end point definitions of MI, MACE, and TLR within the clinical trials are reported in Table 1.

**Table 1:** Randomized Controlled Trials With Long‐Term Follow‐Up Included in Meta‐Analysis

Study	Nordic^10^	BBK1^11^, ^12^	PERFECT^13^	Nordic‐Baltic Bifurcation^14^ IV	BBC1^15^, ^16^	EBC2^17^	Smart‐Strategy^18^	DK Crush II^4^	DK Crush V^5^
Publication year	2013^a^	2015	2015	2015	2016^a^	2016	2016	2017^a^	2017
Participants (n)	413	202	419	446	500	202	258	370	482
Strategy	Provisional vs complex	Provisional vs T stent	Provisional vs crush	Provisional vs complex	Provisional vs complex	Provisional vs culotte	Conservative vs aggressive provisional	DK crush vs provisional	DK crush vs provisional
Primary trial end point	MACE (cardiac death, nonprocedural MI, TVR, ST)	All‐cause death, MI, or TLR	Cardiac death, MI, or TVR	MACE (cardiac death, nonprocedural MI, TLR, and definite ST)	Death	Death, MI, or TVR	TVF: cardiac death, spontaneous MI, or TVR	Cardiac death, MI, or TVR	Cardiac death, target vessel MI, or TLR
Enrollment period	Sep 2004 to May 2005	Apr 2005 to Aug 2006	Apr 2007 to Jan 2013	N/A	Dec 2004 to Dec 2007	Apr 2011 to Jan 2014	Jul 2007 to Dec 2010	Apr 2007 to Jun 2009	Dec 2011 to Feb 2016
Follow‐up duration, y	5	5	1	2	5	1	3	5	1
Mortality adjudication	Hospital records and national mortality tracking	Hospital records/treating physician	N/A	N/A	National mortality records	Outpatient review or teleconsult	National mortality registry	N/A	N/A
Follow‐up angiography, mo (% of patients)	8 (88)	9 (95)	8 (72)	8 (N/A)	9 (N/A)	N/A	9 (84.5)	9 (92)	13 (66)
Loss to follow‐up, %	2	0	<1	<1	0	1	0	<1	0
Mean age, y	63	66.8	61.0	63.5	63	64	62	64.5	64.5
Diabetes mellitus, %	12.5	22.3	27.4	15.8	12.3	28	27.1	31.5	27.2
Smoker,%	N/A	11.9	28.9	20.1	20.7	53	28	N/A	33.2
Male, %	77	78.8	75.2	N/A	77.1	82	82.5	77.5	80.3
ACS presentation, %	34.5	0	38.2	N/A	33.8	31.5	29.5	84.2	82.9 (includes UAP)
Minimum DAPT duration, mo	6	6	12	N/A	9	12	N/A	12	12
LMCA/LAD/Cx, %	0.7	0	0	2	0	0	44.2	16.7	100
LAD/Diag, %	73.5	74.3	93.0	75.4	82	77.5	45.7	59.8	0

ACS indicates acute coronary syndrome; BBC1, British Bifurcation Coronary Study One; BBK1, Bifurcation Bad Krozingen trial; Cx, circumflex artery; DAPT, dual antiplatelet therapy; Diag, diagonal artery; DK Crush II, Double Kissing Crush versus Provisional Stenting Technique for Treatment of Coronary Bifurcation Lesions; DK crush V, Double Kissing Crush Versus Provisional T Stenting Technique for the Treatment of Unprotected Distal Left Main True Bifurcation Lesions; EBC2, European Bifurcation Coronary Two; LAD, left anterior descending artery; LMCA, left main coronary artery; MACE, major adverse cardiac events; MI, myocardial infarction; N/A, not available; Nordic, The NORDIC Bifurcation Study; PERFECT, Optimal Stenting Strategy for True Bifurcation Lesions; Smart‐Strategy, Optimal Strategy for Side Branch Stenting in Coronary Bifurcation Lesions; ST, stent thrombosis; TLR, target lesion revascularization; TVF, target vessel failure; TVR, target vessel revascularization; UAP, unstable angina pectoris.

a 5‐y follow‐up results.

### Statistical Analysis

Weighted mean follow‐up duration was calculated according to study size. Pooled mean data were used to compare procedural aspects between the groups using an unpaired *t* test. All major study hypotheses were prespecified and tested on an intention‐to‐treat basis with a 2‐tailed α of 0.05.

The random‐effects method was selected because of the various studies estimating different yet related intervention effects related to different types of 2‐stent techniques and lesions enrolled. We also performed a fixed‐effect (Mantel–Haenszel) approach for completeness. We summarize the estimate of effect incorporating the clinical outcomes as the risk ratio (RR) with 95% confidence intervals (CIs). Heterogeneity testing was performed with Higgins I^2^ and a threshold of >50% suggestive of significant heterogeneity between studies.^19^ Given that the expected number of events was anticipated to be small, typically used large sample approximations were anticipated to be less reliable. Exact probability assessment with the Fisher exact test was thus also performed. A prespecified sensitivity analysis was performed of studies reporting >3 years of outcome data to determine more information on long‐term outcomes. Statistical analysis was performed using STATA software v13 and RevMan v5. We also performed an explorative metaregression analysis to assess the effect of selected variables (acute coronary syndrome, minimum duration of DAPT, left main bifurcation, final kissing balloon inflation, and stent generation [first‐ or second‐generation drug‐eluting stent]) on all‐cause mortality.

## Results

Our initial search yielded 278 studies. Nine randomized trials met the inclusion criteria, reporting mortality at ≥1 year of follow‐up.^4^, ^5^, ^10^, ^11^, ^13^, ^14^, ^15^, ^16^, ^17^, ^18^

A total of 3265 patients were enrolled with weighted mean follow‐up of 3.1±1.8 years. The total loss to follow‐up of patients in the study was <1%. In total, 1633 were randomized to an initial single‐stent strategy and 1632 were randomized to a 2‐stent strategy. The mean age of the population was 63.4±1.4 years. Overall, 59.9% of the procedures were performed in patients with stable coronary artery disease, whereas 40.1% underwent percutaneous coronary intervention for acute coronary syndrome. Patient demographics are listed in Table 1.

The use of a single‐stent strategy was associated with shorter procedural time (52.2±12.5 versus 64.0±17.0 minutes; *P*<0.001) and less volume of contrast used (227.6±68.7 versus 250.7±66.3 mL; *P*<0.001). Crossover to 2 stents in the single‐stent group occurred in 17.9% of lesions treated. Crossover to a single stent in the 2‐stent group occurred in 7.6% of lesions. Further procedural details are listed in Table 2.

**Table 2:** Procedural Factors in Coronary Bifurcation Trials

Procedural Factors	Nordic^10^ 2013^a^	BBK1^11^, ^12^ 2015	PERFECT^13^ 2015	Nordic‐Baltic Bifurcation^14^ IV 2015	BBC1^15^, ^16^ 2016^a^	EBC2^17^ 2016	Smart‐Strategy^18^ 2016	DK Crush II^4^ 2017^a^	DK Crush V^5^ 2017
True bifurcation, (%, involves SB)	72	68.3	86.6	100	83.2	100	66.3	100	100
Two‐stent technique (%)	Crush (50), culotte (21), other (29)	T‐stent	Crush (99)	Culotte (67), T‐stenting (7), other (26)	Crush (68), culotte (30), other (2)	Culotte	TAP	DK Crush	DK Crush
Stent type (%)	SES	SES	SES (58), EES (28), ZES (9), other (5)	SES (50), EES (50)	PES	BES (100)	SES (47), EES (29), other (24)	Excel (rapamycin‐eluting stents with biodegradeable polymer)	N/A
FKB (%, simple/complex)	32/74	100/100	79/96	36.1/91.2	29/76	94/96	26/69	79.2/100	78.9/99.6
IVUS	N/A	N/A	95.7	N/A	N/A	N/A	97.7	47.0	41.7
Procedure time (min, simple/complex)	62/76	51/56	49/54	N/A	57/78	23/39	N/A	37/38	66/82
Contrast, mL	233/283	204/203	347/350	N/A	N/A	246/269	N/A	137/149	191/227
SB stent (min, simple/complex)	4.4/95	18.8/97	28.2/97.7	3.7/96	2.8/98	16/97	7/30	29/100	47.1/100
MV lesion length, mm	16	21.3	28.4	N/A	N/A	18	N/A	27.1	48.7
SB lesion length, mm	5	10.2	9.3	5.8	N/A	10.3	N/A	15.0	30.4
SB diameter stenosis, %	N/A	53.8	55.2	45.8	N/A	54.5	N/A	N/A	65.6

BES indicates biolimus‐eluting stent; DK, double‐kissing; EES, everolimus‐eluting stent; FKB, final kissing balloon; IVUS, intravascular ultrasound; MV, main vessel; N/A, not available; PES, paclitaxel‐eluting stent; SB, side branch; SES, sirolimus‐eluting stent; TAP, T stenting and small protrusion; ZES, zotarolimus‐eluting stent.

a 5 year follow‐up results.

**Figure 2:**
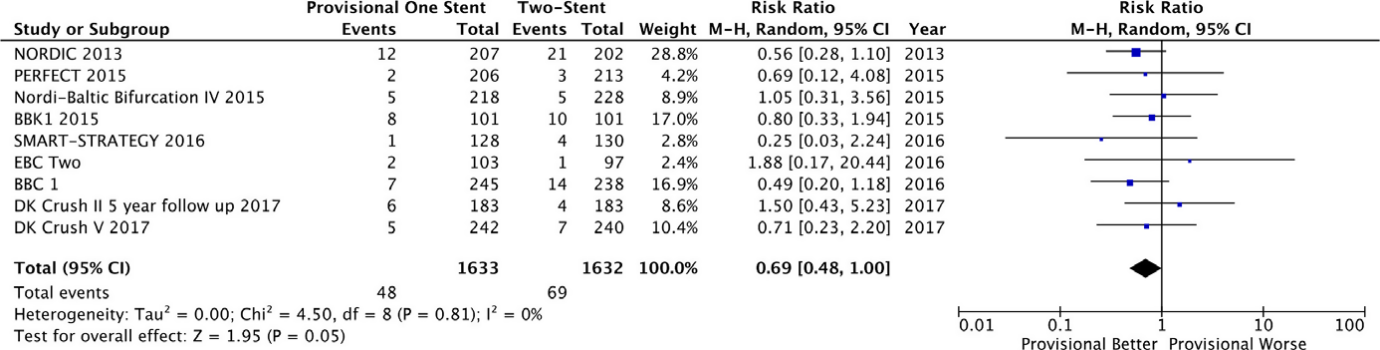
Forest plot of primary outcome: all‐cause mortality according to bifurcation treatment strategy. Nordic 2013,^10^ BBK,^11^, ^12^ PERFECT,^13^ Nordic‐Baltic Bifurcation IV,^14^ BBC1,^15^, ^16^ EBC2,^17^ Smart‐Strategy,^18^ DK Crush II 2017,^4^ DK Crush V.^5^ CI indicates confidence interval; M‐H, Mantel‐Haenszel test.

**Figure 3:**
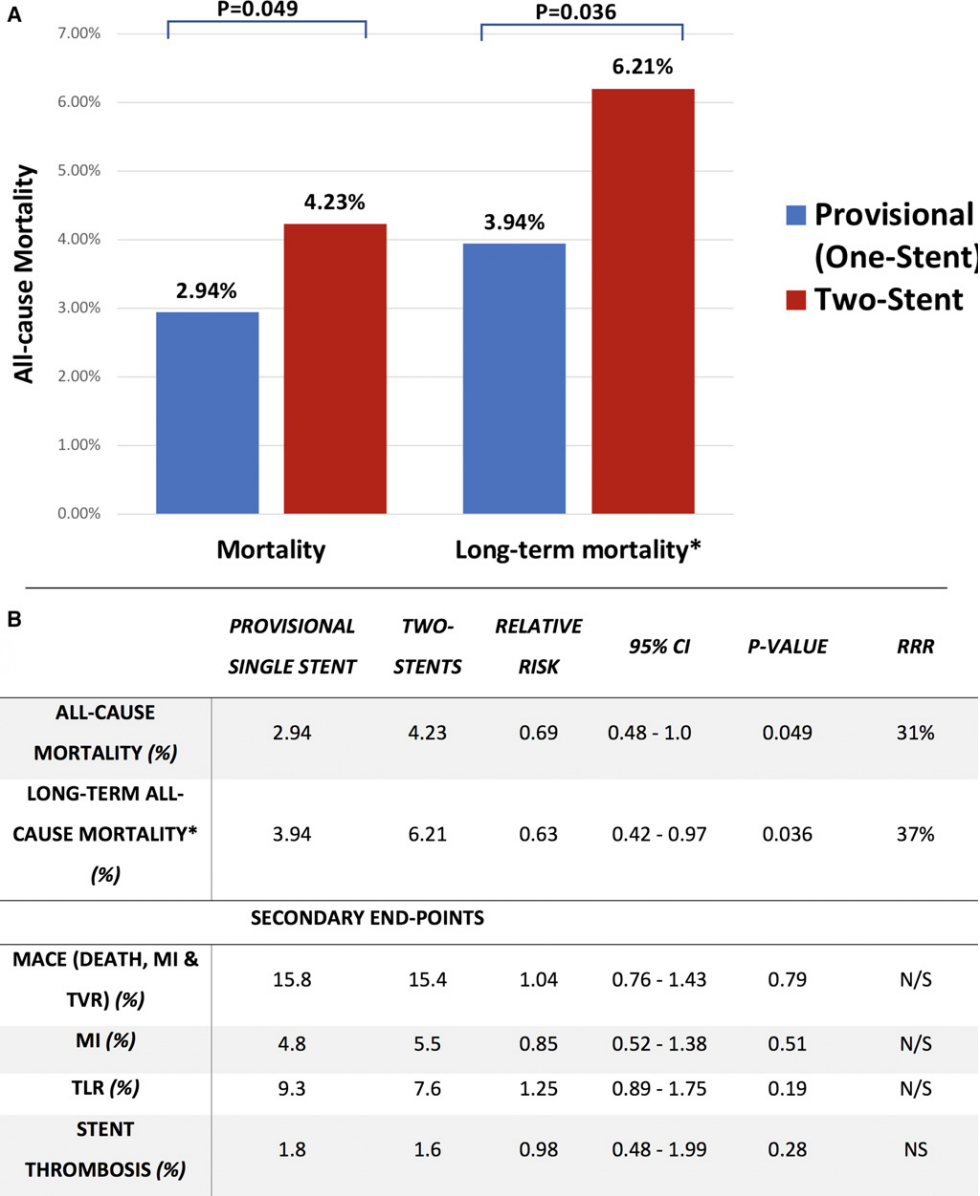
A, All‐cause mortality according to bifurcation treatment strategy. 3265 patients were enrolled in 9 randomized trials with weighted mean follow‐up of 3.1 years. The total loss to follow‐up of patients in the study was < 1%. Nordic 2013,^10^ BBK,^11^, ^12^ PERFECT,^13^ Nordic‐Baltic Bifurcation IV,^14^ BBC1,^15^, ^16^ EBC2,^17^ Smart‐Strategy,^18^ DK Crush II 2017,^4^ DK Crush V.^5^ B, Long‐term health outcomes of provisional stenting vs a complex strategy for treatment of coronary bifurcation lesions. *Long‐term mortality incorporating trials with ≥3 years of clinical follow‐up: A prespecified sensitivity analysis was carried out of five studies including 1713 patients followed up for an average of 4.7 years. DK CrushII,^4^ Nordic,^10^ BBK,^12^ BBC1,^15^ Smart‐Strategy.^18^ CI indicates confidence interval; MACE, major adverse cardiac events; MI, myocardial infarction; RRR, relative risk reduction with provisional vs 2‐stent strategies; TLR, target lesion revascularization.

Compared with 2‐stent techniques, the provisional single‐stent approach was associated with lower all‐cause mortality (2.94% versus 4.23%; RR=0.69; 95% CI, 0.48–1.00; 2‐sided Fisher exact test, *P*=0.049; I^2^=0%; Figures 2 and 3). The absolute risk difference in mortality was 1.29% lower with a provisional single‐stent technique (95% CI, 0.01–2.56%; 2‐sided Fisher exact test, *P*=0.049). An alternative fixed‐effects analysis provided similar results (2.94% versus 4.23%; RR=0.69; 95% CI, 0.48–0.99; *P*=0.04).

**Figure 4:**
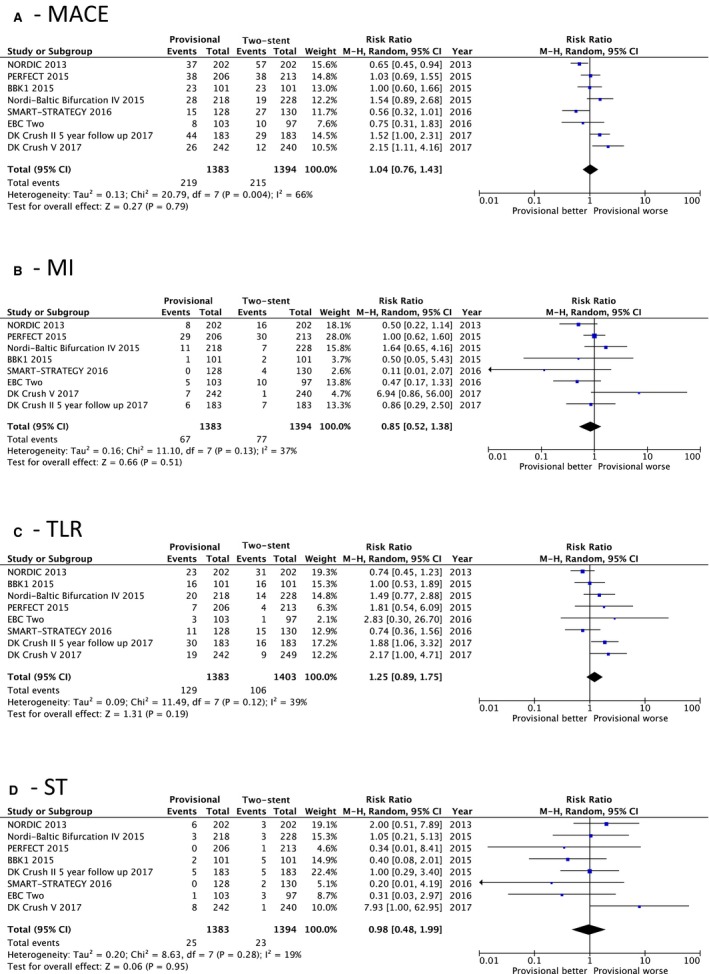
Forest plots for secondary end points. A, Major adverse cardiac events (MACE). B, Myocardial infarction (MI). C, Target lesion revascularization (TLR). D, Stent thrombosis (ST).

There was no difference in MACE (15.8% versus 15.4%; RR=1.04; 95% CI, 0.76–1.43; *P*=0.79; I^2^=66%) or MI (4.8% versus 5.5%; RR=0.85; 95% CI, 0.52–1.38; *P*=0.51; I^2^=37%) between the allocated treatment groups. These secondary end points at ≥12 months of follow‐up were reported for 8 of the 9 RCTs in the analysis. Further secondary end points are plotted in Figure 4, confirming no difference in TLR (9.3% versus 7.6%; RR=1.25; 95% CI, 0.89–1.75; *P*=0.19; I^2^=39%) or ST (1.8% versus 1.6%; RR=0.98; 95% CI, 0.38–1.99; *P*=0.28; I^2^=19%).

**Figure 5:**
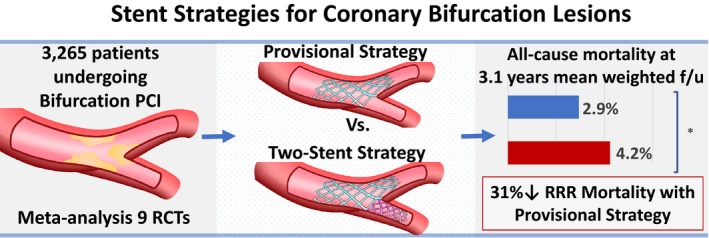
Central illustration (graphic summary of study). f/u indicates follow‐up; PCI, percutaneous coronary intervention; RCTs, randomized controlled trials; RRR, relative risk ratio. *Denotes *P*=0.049

A prespecified sensitivity analysis was carried out to investigate only trials with ≥3 years of follow‐up. Five studies were included, with a total of 1713 patients followed up for an average of 4.7 years.^4^, ^10^, ^12^, ^15^, ^18^ Compared with an up‐front 2‐stent technique, stenting the MV only was associated with a lower risk of all‐cause mortality (3.9% versus 6.2%; RR 0.63; 95% CI, 0.42–0.97; *P*=0.036; I^2^=0%) with a trend toward a reduction in MI (2.4% versus 4.7%; RR=0.56; 95% CI, 0.3–1.04; *P*=0.06; I^2^=0%). There were no significant differences in long‐term TLR or ST. Figure 3 provides the main outcomes of interest in the study. Metaregression analysis did not show any significant relationship between primary outcome effect size and selected clinical variables (eg, final kissing balloon inflation, proportion of first‐generation drug‐eluting stent use, or left main coronary bifurcation location). A summary of this study is shown in the central illustration (Figure 5).

## Discussion

In the largest meta‐analysis of randomized trials comparing treatment strategies for coronary bifurcation lesions, we have shown reduced all‐cause mortality at medium‐ to long‐term follow‐up in patients randomly assigned to an initial strategy of MV stenting only compared with up‐front stenting of both the MV and SB. There was a 31% relative risk reduction in death at mean weighted follow‐up of 3.1 years, extending to an overall 37% relative risk reduction at mean 4.7 years of follow‐up. The provisional single‐stent “less is more” approach is attractive and clinically impactful because of simplicity without compromising long‐term outcomes. Overall, long‐term all‐cause mortality is low throughout the studies. We included in our analysis the patient‐level 5‐year data from the Nordic bifurcation study and BBC1 (British Bifurcation Coronary Study) in which reduced mortality with the single‐stent approach was previously reported.^15^ It is plausible that the use of first‐generation drug‐eluting stents in these 2 studies led to increased events in the 2‐stent cohorts compared with later trials using more contemporary devices.

Notably, we did not find a difference in MACE, MI, TLR, or ST; therefore, the reason for the increased death rate is not immediately apparent. There are several reasons why this might be seen. One limiting assumption is that the differential mortality rates most likely reflect increased cardiac death from STs. Most studies considered all‐cause mortality rather than specific cardiac mortality. Another potentially relevant mode of all‐cause death in the 2‐stent group relates to bleeding episodes, which would vary according to the duration of DAPT. Physicians treating patients in these open‐label trials may preferentially keep the 2‐stent group on prolonged DAPT with inherent risks of bleeding related morbidity, and mortality in the long term. Indeed, a large meta‐analysis of RCTs showed increased all‐cause mortality in patients randomized to extended DAPT (odds ratio: 1.30; 95% CI, 1.02–1.66; *P*=0.03).^20^ Bleeding events and duration of DAPT were not uniformly reported, so the precise cause of mortality is unclear. Although all‐cause mortality reduction can be a challenging primary end point to meet for an individual RCT, it has some theoretical advantages when used in a meta‐analysis with long‐term follow‐up. It is a binary outcome that is easy to determine and of utmost clinical important to patients, trial designers, and funders. All‐cause mortality is a robust measure, particularly given declining rates of nonforensic autopsy—sudden death in elderly patients is not usually investigated with a postmortem.^21^ Academic Research Consortium criteria for acute ST may not be met despite autopsy‐proven ST. This suggests that ST is likely to be substantially underreported.^22^ Including all‐cause mortality improves the power to detect these important events and is the most reliably obtained clinical outcome at long‐term follow‐up.

Data from nonrandomized trials also support the more conservative approach of MV stenting only.^23^ Our work builds on a previous meta‐analysis^24^ by incorporating 2 recent seminal publications with long‐term follow‐up supporting the DK crush 2‐stent bifurcation technique.^4^, ^5^ Despite the inclusion of these data sets, the provisional stent strategy still appears to be associated with a reduced mortality at medium‐ to long‐term follow‐up.

After 5 years of follow‐up in the DK Crush II (Double Kissing Crush versus Provisional Stenting Technique for Treatment of Coronary Bifurcation Lesions) trial, patients treated with the 2‐stent strategy had improved outcomes compared with MV‐only stenting. This effect was largely driven by reduced TLR. After 1 year of follow‐up in the DK Crush V (Double Kissing and Double Crush Versus Provisional T Stenting Technique for the Treatment of Unpro‐ tected Distal Left Main True Bifurcation Lesions: A Randomized, International, Multi‐Center Clinical Trial) study, patients with left main bifurcation lesions treated with the 2‐stent strategy had a reduction in MACE, ST, and TLR compared with MV stenting only. DK Crush V was the only study to exclusively enroll patients with left main lesions—these patients had complex and extensive SB involvement (mean diameter stenosis 66%, lesion length 30 mm). Many operators would not feel comfortable adopting a provisional strategy for such complex disease involving a large SB. It is important to acknowledge that the DK crush technique is not simple, and the trial findings may not be generalizable to the typical interventional cardiologist. In DK Crush V, operators had to perform at least 300 percutaneous coronary interventions per year for 5 consecutive years to recruit patients into the study. In addition, they had to demonstrate proficiency of the technique by submitting 5 exemplary cases of DK crush to the investigators before taking part. Importantly, the DK crush studies may account for the heterogeneity observed in secondary end points in the meta‐analysis. Differences between these and other studies include more patients with acute coronary syndrome (pooled mean: 83.4% versus 30.7%) and left main coronary disease (63.8% versus 5.2%), as well as more frequent stenting of the SB in the provisional stent group (39.2% versus 10.6%). Furthermore, there was increased use of final kissing balloons in the single‐stent arm (79.0% versus 50.3%). The high crossover to 2 stents in the patients randomized to single stent may reflect an increased complexity of bifurcation disease in the DK crush trials and may explain the surprisingly high rate of ST in the provisional arm of DK Crush V (3.3% versus 0.4%; *P*=0.02).

### Limitations

A significant proportion of trials in our analysis used first‐generation drug‐eluting stents (n=4). These stents have a known higher and continuous risk of ST for over 5 years and may have driven excess mortality in the up‐front 2‐stent group.^25^ Metaregression incorporating the percentage of first‐generation drug‐eluting stents in each trial did not show a statistically significant interaction with effect size. Nevertheless, our conclusions may not be generalizable to contemporary practice using newer generation drug‐eluting stents.

Despite the prespecified nature of our sensitivity analysis, many secondary analyses were performed at the 5% level of significance without any adjustments for multiple testing. Furthermore, Higgins I^2^ suggested significant heterogeneity of secondary outcome measures in the meta‐analysis. Methodology, demographics, and definitions of MI, MACE, and TLR varied between studies and may account for some of this (Table 1). Differences in bifurcation lesion complexity, anatomical location, 2‐stent techniques, procedural details, stent types, and intensity of DAPT are all likely to be present but could not be fully accounted for (Table 2). Most trials had follow‐up coronary angiography mandated in the protocol. This was done typically 8 to 9 months after the index procedure, occurring before the adjudication of 12‐month outcomes including target vessel revascularization. The oculostenotic reflex and related revascularization is a known pitfall of routine angiographic follow‐up and may have driven events in this study.^26^ Individual patient‐level data were not available but would have been helpful to probe these factors. We included the SMART‐STRATEGY trial (optimal strategy for SB stenting in coronary bifurcation lesion) although the rate of SB stenting in the 2‐stent arm was <50%. The ongoing EBC main trial aims to determine whether the approach of stenting the MV only is safe and effective in the contemporary treatment of true left main bifurcation lesions.^27^ Although our study supports the simplicity of a provisional single‐stent approach, operators may reasonably opt for an up‐front 2‐stent strategy in patients with complex bifurcation lesions with extensive involvement of a large SB (particularly left main disease).

## Conclusion

Despite recent studies supporting a default 2‐stent strategy for treatment of coronary bifurcation lesions, no one size fits all. Our meta‐analysis supports the provisional single‐stent strategy as the default approach for treatment of coronary bifurcation lesions.

## Author Contributions

T.F. devised and wrote the first draft and performed statistical analysis. T.F. and P.M. performed the systematic review, and T.F. and D.C. extracted the data for analysis. D.C.O.L. and B.H. checked the data and provided consensus. M.M.E., P.R., D.H.S., K.G.O., & C.B. devised aspects of the study protocol, provided critical manuscript appraisal and edited the final article.

## Sources of Funding

This work was supported by the British Heart Foundation (BHF) (RE/13/5/30177; PG/17/25/32884).

## Disclosures

Berry is employed by the University of Glasgow, which holds consultancy and research agreements with companies that have commercial interests in the diagnosis and treatment of angina. The companies include Abbott Vascular, AstraZeneca, Boehringer Ingelheim, Menarini Pharmaceuticals, and Siemens Healthcare. Oldroyd has received consultant and speaker fees from Abbott Vascular which manufacture pressure wires. None of these companies have had any involvement with this study. None of the other authors have any potential conflicts of interest.

## References

[1] Louvard Y , Thomas M , Dzavik V , Hildick‐Smith D , Galassi AR , Pan M , Burzotta F , Zelizko M , Dudek D , Ludman P , Sheiban I , Lassen JF , Darremont O , Kastrati A , Ludwig J , Iakovou I , Brunel P , Lansky A , Meerkin D , Legrand V , Medina A , Lefevre T . Classification of coronary artery bifurcation lesions and treatments: time for a consensus!. Catheter Cardiovasc Interv. 2008;71:175–183. 17985377 10.1002/ccd.21314

[2] Palmerini T , Sangiorgi D , Marzocchi A , Tamburino C , Sheiban I , Margheri M , Vecchi G , Sangiorgi G , Ruffini M , Bartorelli AL , Briguori C , Vignali L , Di Pede F , Ramondo A , Inglese L , De Carlo M , Bolognese L , Benassi A , Palmieri C , Filippone V , Barlocco F , Lauria G , De Servi S . Ostial and midshaft lesions vs. bifurcation lesions in 1111 patients with unprotected left main coronary artery stenosis treated with drug‐eluting stents: results of the survey from the Italian Society of Invasive Cardiology. Eur Heart J. 2009;30:2087–2094. 19508996 10.1093/eurheartj/ehp223

[3] Brar SS , Gray WA , Dangas G , Leon MB , Aharonian VJ , Brar SK , Moses JW . Bifurcation stenting with drug‐eluting stents: a systematic review and meta‐analysis of randomised trials. EuroIntervention. 2009;5:475–484. 19755337 10.4244/eijv5i4a76

[4] Chen SL , Santoso T , Zhang JJ , Ye F , Xu YW , Fu Q , Kan J , Zhang FF , Zhou Y , Xie DJ , Kwan TW . Clinical Outcome of Double Kissing Crush Versus Provisional Stenting of Coronary Artery Bifurcation Lesions: The 5‐Year Follow‐Up Results From a Randomized and Multicenter DKCRUSH‐II Study (Randomized Study on Double Kissing Crush Technique Versus Provisional Stenting Technique for Coronary Artery Bifurcation Lesions). Circ Cardiovasc Interv. 2017;10:e004497. 28122805 10.1161/CIRCINTERVENTIONS.116.004497 PMC5319391

[5] Chen SL , Zhang JJ , Han Y , Kan J , Chen L , Qiu C , Jiang T , Tao L , Zeng H , Li L , Xia Y , Gao C , Santoso T , Paiboon C , Wang Y , Kwan TW , Ye F , Tian N , Liu Z , Lin S , Lu C , Wen S , Hong L , Zhang Q , Sheiban I , Xu Y , Wang L , Rab TS , Li Z , Cheng G , Cui L , Leon MB , Stone GW . Double kissing crush versus provisional stenting for left main distal bifurcation lesions: DKCRUSH‐V randomized trial. J Am Coll Cardiol. 2017;70:2605–2617. 29096915 10.1016/j.jacc.2017.09.1066

[6] Moher D , Shamseer L , Clarke M , Ghersi D , Liberati A , Petticrew M , Shekelle P , Stewart LA ; Group P‐P . Preferred reporting items for systematic review and meta‐analysis protocols (PRISMA‐P) 2015 statement. Syst Rev. 2015;4:1. 25554246 10.1186/2046-4053-4-1 PMC4320440

[7] Higgins JP , Altman DG , Gotzsche PC , Juni P , Moher D , Oxman AD , Savovic J , Schulz KF , Weeks L , Sterne JA ; Cochrane Bias Methods G and Cochrane Statistical Methods G . The Cochrane Collaboration's tool for assessing risk of bias in randomised trials. BMJ. 2011;343:d5928. 22008217 10.1136/bmj.d5928 PMC3196245

[8] Sterne JAC , Egger M . Funnel plots for detecting bias in meta‐analysis. J Clin Epidemiol. 2001;54:1046–1055. 11576817 10.1016/s0895-4356(01)00377-8

[9] Mauri L , Hsieh WH , Massaro JM , Ho KK , D'Agostino R , Cutlip DE . Stent thrombosis in randomized clinical trials of drug‐eluting stents. N Engl J Med. 2007;356:1020–1029. 17296821 10.1056/NEJMoa067731

[10] Maeng M , Holm NR , Erglis A , Kumsars I , Niemela M , Kervinen K , Jensen JS , Galloe A , Steigen TK , Wiseth R , Narbute I , Gunnes P , Mannsverk J , Meyerdierks O , Rotevatn S , Nikus K , Vikman S , Ravkilde J , James S , Aaroe J , Ylitalo A , Helqvist S , Sjogren I , Thayssen P , Virtanen K , Puhakka M , Airaksinen J , Christiansen EH , Lassen JF , Thuesen L ; Nordic‐Baltic Percutaneous Coronary Intervention Study G . Long‐term results after simple versus complex stenting of coronary artery bifurcation lesions: Nordic Bifurcation Study 5‐year follow‐up results. J Am Coll Cardiol. 2013;62:30–34. 23644088 10.1016/j.jacc.2013.04.015

[11] Ferenc M , Gick M , Kienzle RP , Bestehorn HP , Werner KD , Comberg T , Kuebler P , Buttner HJ , Neumann FJ . Randomized trial on routine vs. provisional T‐stenting in the treatment of de novo coronary bifurcation lesions. Eur Heart J. 2008;29:2859–2867. 18845665 10.1093/eurheartj/ehn455 PMC2638653

[12] Ferenc M , Ayoub M , Buttner HJ , Gick M , Comberg T , Rothe J , Valina CM , Hochholzer W , Neumann FJ . Long‐term outcomes of routine versus provisional T‐stenting for de novo coronary bifurcation lesions: five‐year results of the Bifurcations Bad Krozingen I study. EuroIntervention. 2015;11:856–859. 26696453 10.4244/EIJV11I8A175

[13] Kim YH , Lee JH , Roh JH , Ahn JM , Yoon SH , Park DW , Lee JY , Yun SC , Kang SJ , Lee SW , Lee CW , Seung KB , Shin WY , Lee NH , Lee BK , Lee SG , Nam CW , Yoon J , Yang JY , Hyon MS , Lee K , Jang JS , Kim HS , Park SW , Park SJ . Randomized comparisons between different stenting approaches for bifurcation coronary lesions with or without side branch stenosis. JACC Cardiovasc Interv. 2015;8:550–560. 25907082 10.1016/j.jcin.2015.01.016

[14] Kumsars I , Niemelä M , Erglis A , Kervinen K , Christiansen EH , Maeng M , Dombrovskis A , Abraitis V , Kibarskis A , Steigen TK , Trovik T , Latkovskis G , Sondore D , Narbute I , Terkelsen CJ , Eskola M , Romppanen H , Thayssen P , Kaltoft A , Vasankari T , Gunnes P , Frobert O , Calais F , Hartikainen J , Jensen SE , Engstrøm T , Holm NR , Lassen JF , Thuesen L . Randomized comparison of provisional side branch stenting versus a two‐stent strategy for treatment of true coronary bifurcation lesions involving a large side branch. Two‐year results in the Nordic‐Baltic bifurcation study IV. Paper presented at: EuroPCR 2015; Paris. 10.1136/openhrt-2018-000947 PMC6999681 32076558

[15] Behan MW , Holm NR , de Belder AJ , Cockburn J , Erglis A , Curzen NP , Niemela M , Oldroyd KG , Kervinen K , Kumsars I , Gunnes P , Stables RH , Maeng M , Ravkilde J , Jensen JS , Christiansen EH , Cooter N , Steigen TK , Vikman S , Thuesen L , Lassen JF , Hildick‐Smith D . Coronary bifurcation lesions treated with simple or complex stenting: 5‐year survival from patient‐level pooled analysis of the Nordic Bifurcation Study and the British Bifurcation Coronary Study. Eur Heart J. 2016;37:1923–1928. 27161619 10.1093/eurheartj/ehw170

[16] Hildick‐Smith D , de Belder AJ , Cooter N , Curzen NP , Clayton TC , Oldroyd KG , Bennett L , Holmberg S , Cotton JM , Glennon PE , Thomas MR , Maccarthy PA , Baumbach A , Mulvihill NT , Henderson RA , Redwood SR , Starkey IR , Stables RH . Randomized trial of simple versus complex drug‐eluting stenting for bifurcation lesions: the British Bifurcation Coronary Study: old, new, and evolving strategies. Circulation. 2010;121:1235–1243. 20194880 10.1161/CIRCULATIONAHA.109.888297

[17] Hildick‐Smith D , Behan MW , Lassen JF , Chieffo A , Lefevre T , Stankovic G , Burzotta F , Pan M , Ferenc M , Bennett L , Hovasse T , Spence MJ , Oldroyd K , Brunel P , Carrie D , Baumbach A , Maeng M , Skipper N , Louvard Y . The EBC TWO Study (European Bifurcation Coronary TWO): A Randomized Comparison of Provisional T‐Stenting Versus a Systematic 2 Stent Culotte Strategy in Large Caliber True Bifurcations. Circ Cardiovasc Interv. 2016;9:e003643. DOI: 10.1161/CIRCINTERVENTIONS.115.003643. 10.1161/CIRCINTERVENTIONS.115.003643 27578839

[18] Song YB , Park TK , Hahn JY , Yang JH , Choi JH , Choi SH , Lee SH , Gwon HC . Optimal strategy for provisional side branch intervention in coronary bifurcation lesions: 3‐year outcomes of the SMART‐STRATEGY randomized trial. JACC Cardiovasc Interv. 2016;9:517–526. 27013152 10.1016/j.jcin.2015.11.037

[19] Higgins JP , Thompson SG , Deeks JJ , Altman DG . Measuring inconsistency in meta‐analyses. BMJ. 2003;327:557–560. 12958120 10.1136/bmj.327.7414.557 PMC192859

[20] Navarese EP , Andreotti F , Schulze V , Kolodziejczak M , Buffon A , Brouwer M , Costa F , Kowalewski M , Parati G , Lip GY , Kelm M , Valgimigli M . Optimal duration of dual antiplatelet therapy after percutaneous coronary intervention with drug eluting stents: meta‐analysis of randomised controlled trials. BMJ. 2015;350:h1618. 25883067 10.1136/bmj.h1618 PMC4410620

[21] Shojania KG , Burton EC . The vanishing nonforensic autopsy. N Engl J Med. 2008;358:873–875. 18305264 10.1056/NEJMp0707996

[22] Cutlip DE , Nakazawa G , Krucoff MW , Vorpahl M , Mehran R , Finn AV , Vranckx P , Kimmelstiel C , Berger C , Petersen JL , Palabrica T , Virmani R . Autopsy validation study of the academic research consortium stent thrombosis definition. JACC Cardiovasc Interv. 2011;4:554–559. 21596329 10.1016/j.jcin.2011.01.011

[23] Spaziano M , Roy A , Akodad M , Louvard Y , Lefevre T , Serruys P , Chevalier B , Morice MC . Five‐year outcomes of bifurcation stenting: insights from the SYNTAX trial. Eur Heart J. 2017;38:443–444.

[24] Nairooz R , Saad M , Elgendy IY , Mahmoud AN , Habash F , Sardar P , Anderson D , Shavelle DM , Abbott JD . Long‐term outcomes of provisional stenting compared with a two‐stent strategy for bifurcation lesions: a meta‐analysis of randomised trials. Heart. 2017;103:1427–1434. 28314731 10.1136/heartjnl-2016-310929

[25] Yamaji K , Raber L , Zanchin T , Spitzer E , Zanchin C , Pilgrim T , Stortecky S , Moschovitis A , Billinger M , Schonenberger C , Eberli F , Juni P , Luscher TF , Heg D , Windecker S . Ten‐year clinical outcomes of first‐generation drug‐eluting stents: the Sirolimus‐Eluting vs. Paclitaxel‐Eluting Stents for Coronary Revascularization (SIRTAX) VERY LATE trial. Eur Heart J. 2016;37:3386–3395. 27578808 10.1093/eurheartj/ehw343

[26] Shiomi H , Morimoto T , Kitaguchi S , Nakagawa Y , Ishii K , Haruna Y , Takamisawa I , Motooka M , Nakao K , Matsuda S , Mimoto S , Aoyama Y , Takeda T , Murata K , Akao M , Inada T , Eizawa H , Hyakuna E , Awano K , Shirotani M , Furukawa Y , Kadota K , Miyauchi K , Tanaka M , Noguchi Y , Nakamura S , Yasuda S , Miyazaki S , Daida H , Kimura K , Ikari Y , Hirayama H , Sumiyoshi T , Kimura T , ReACT Investigators . The ReACT trial: randomized evaluation of routine follow‐up coronary angiography after percutaneous coronary intervention trial. JACC Cardiovasc Interv. 2017;10:109–117. 28040445 10.1016/j.jcin.2016.10.018

[27] Chieffo A , Hildick‐Smith D . The European Bifurcation Club Left Main Study (EBC MAIN): rationale and design of an international, multicentre, randomised comparison of two stent strategies for the treatment of left main coronary bifurcation disease. EuroIntervention. 2016;12:47–52. 27173861 10.4244/EIJV12I1A8

